# Single origin of the Mascarene stick insects: ancient radiation on sunken islands?

**DOI:** 10.1186/s12862-015-0478-y

**Published:** 2015-09-16

**Authors:** Sven Bradler, Nicolas Cliquennois, Thomas R. Buckley

**Affiliations:** Johann-Friedrich-Blumenbach-Institute of Zoology and Anthropology, Georg-August-University Göttingen, Berliner Str. 28, 37073 Göttingen, Germany; Collège français, Lot 02 F 15 Tomboarivo, B.P. 141, 110, Antsirabe, Madagascar; Landcare Research, Private Bag 92170, Auckland, New Zealand; School of Biological Sciences, The University of Auckland, Private Bag 92019, Auckland, New Zealand; Allan Wilson Centre, Auckland, New Zealand

## Abstract

**Background:**

The study of islands as model systems plays a key role in understanding many evolutionary processes. Knowledge of the historical events leading to present-day island communities is pivotal for exploring fundamental mechanisms of speciation and adaptation. The remote Mascarene archipelago (Mauritius, Réunion, Rodrigues), considered to be the product of an age-progressive trend of north-to-south volcanic activity in the Indian Ocean, hosts a remarkably diverse, endemic and threatened concentration of flora and fauna that has traditionally been considered to be biogeographically related to Madagascar and Africa. To explore the evolutionary diversity of the Mascarene stick insects (Phasmatodea), we constructed a global phylogeny from approximately 2.4 kb of mitochondrial and nuclear sequence data of more than 120 species representing all major phasmatodean lineages.

**Results:**

Based on the obtained time-calibrated molecular tree we demonstrate that the current phasmid community of the Mascarene archipelago, which consists of members of four presumably unrelated traditional subfamilies, is the result of a single ancient dispersal event from Australasia and started radiating between 16–29 million years ago, significantly predating the age of Mauritius (8–10 million years).

**Conclusions:**

We propose that the Mascarene stick insects diversified on landmasses now eroded away, presumably to the north of Mauritius. In consequence, ancient islands have probably persisted in the Indian Ocean until the emergence of Mauritius and not only served as stepping stones for colonisation events during sea-level lowstands, but as long-lasting cradles of evolution. These ancient landmasses most likely allowed for adaptive speciation and served as significant sources of diversity that contributed to the biomes of the Mascarene archipelago and the megadiverse Madagascar.

**Electronic supplementary material:**

The online version of this article (doi:10.1186/s12862-015-0478-y) contains supplementary material, which is available to authorized users.

## Background

The Indian Ocean harbours a number of major terrestrial biodiversity hotspots including Madagascar and nearby archipelagos of the Comores, Seychelles and Mascarenes [1]. While Madagascar and the granitic Seychelles are of old continental origins, the Mascarene Islands Mauritius and Réunion have been shown to be much younger as the result of the similar process of plate movement over a stationary volcanic hot spot that formed the Hawaiian archipelago and the Galápagos Islands in the Pacific [2–5]. Recent studies suggest the presence of Precambrian, albeit currently submerged continental fragments underneath the Mascarene Plateau covered by magmatic deposits in the Indian Ocean that separated from Madagascar about 61–83.5 million years ago (mya) [6]. Although less well-studied, biologically speaking, than the Hawaiian and Galápagos Islands, the Mascarene archipelago appears to be likewise rich in biodiversity, notably in endemics [7]. Unfortunately, the Mascarene Islands, Mauritius and Rodrigues in particular, have already lost a significant amount of this endemic diversity since human settlement in the 17^th^ century due to habitat destruction [7, 8]. Understanding the biogeographic processes leading to endemic diversity is crucial both to interpretation of their evolutionary history and to the establishment of conservation strategies [9]. The majority of the extant species diversity of the Mascarene Islands has been described as the result of recent colonisations from outside the archipelago and divergence within the contemporary island system, often with immigrating lineages derived from Madagascar and Africa [10–14]. Yet, unexpectedly, numerous faunal elements are related to more remote regions such as Asia and the Indo-Pacific [7, 15–18]. In some taxonomic groups like skinks and stick insects, colonisations of the Mascarenes appear to have involved long-distance dispersals from the Australian region ranging between 5600–7000 km [19, 20].

**Fig. 1:**
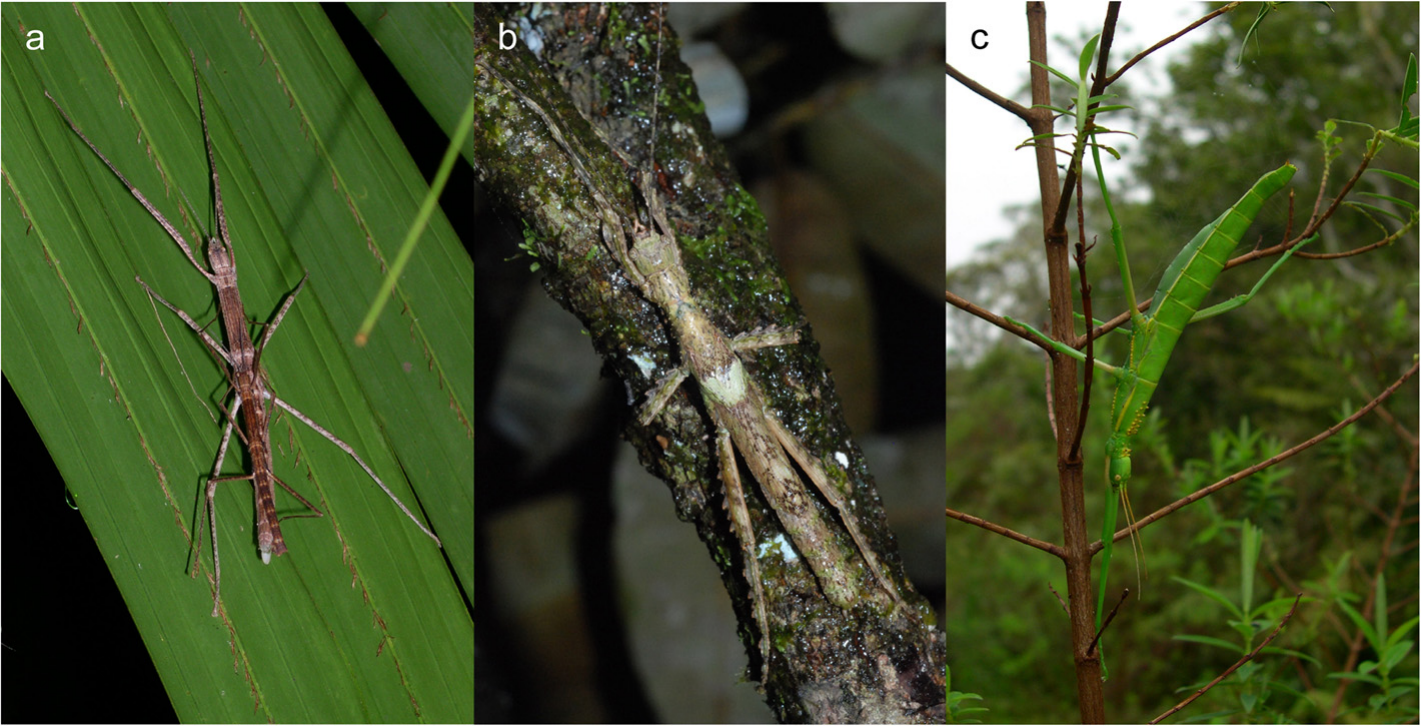
Photos of Mascarene stick insects. **a** Couple of *Apterograeffea reunionensis* (Platycraninae) from Réunion. **b** Male of *Epicharmus marchali* (Xeroderinae) from Mauritius. **c** Female of *Rhaphiderus spiniger* (Tropidoderinae) from Réunion

Stick and leaf insects (insect order Phasmatodea) are particularly informative organisms for historical biogeographic research due to their limited dispersal capabilities, manageable species numbers and ease of sampling. Phasmatodeans are predominantly large nocturnal herbivores, exhibiting extreme forms of plant mimicry (Fig. 1), with very limited flight capability. Stick insects must be considered as being non-volant although they often possess wings, which they generally use to control descent from the canopy rather than for sustained flying [21]. The Mascarene islands harbour stick insects from four traditional subfamilies and one taxon incertae sedis [22, 23], suggesting repeated transoceanic colonisations of the archipelago by unrelated phasmatodean lineages. However, previous studies demonstrated that geographical distribution rather than traditional classification and morphological similarity reflects the evolutionary relationships among stick and leaf insects [20, 24]. Here we provide another example of an unexpected adaptive radiation of stick insects in geographical isolation and that furthermore, the age of this radiation predates the origin of the islands that these species currently inhabit.

## Results

**Fig. 2:**
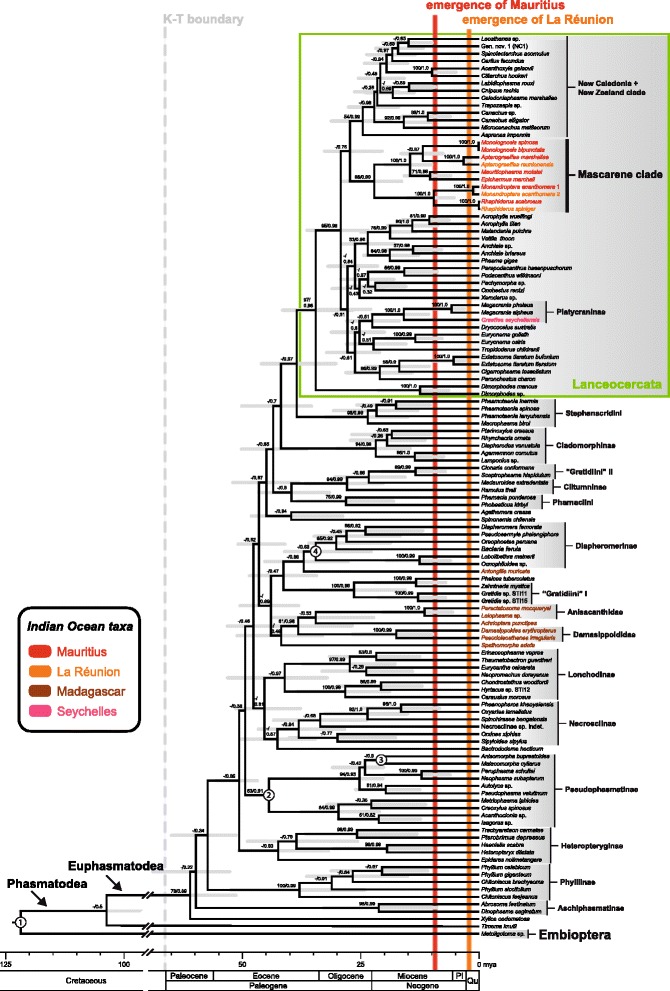
Chronogram of the sampled stick insect specimens with taxa distributed across the Indian Ocean highlighted in tones of red. Numbers at nodes indicate bootstrap values (*left*) and clade posterior probabilities (*right*); *grey bars* show 95 % highest probability density. *Circled numbers* refer to fossil calibration points: (1) *Renphasma* [56], (2) *Eophasma* [61], (3) fossil *Malacomorpha* [63], (4) fossil *Clonistria* [63]. *Pl* Pliocene, *Qu* Quaternary

This study represents the most comprehensive study addressing the global phylogeny of stick and leaf insects to date and the first one to include a representative sampling from landmasses of the Indian Ocean, e.g., from Madagascar and the Mascarene archipelago. We sampled ~2.4 kb of nuclear and mitochondrial sequence data from 121 phasmatodean individuals (representing 120 spp.) and 1 individual (*Metoligotoma*) of the outgroup Embioptera (webspinners). The relaxed clock model placed the root on the branch between the Embioptera and Phasmatodea (Fig. 2), consistent with previous insect phylogenies [25–29]. The next split was between *Timema* and the Euphasmatodea, again consistent with previous phylogenetic reconstructions of the Phasmatodea [25, 30]. The recovered phylogeny was consistent with current phylogenetic hypotheses on stick and leaf insects including some ambiguities at deeper nodes, e.g., the radiation of major phasmatodean lineages, which are currently poorly resolved [24, 31, 32]. We observed strong support for several monophyletic groups, including Aschiphasmatinae (Maximum likelihood bootstrap [MLB] = 98, Bayesian posterior probability [BPP] = 0.99), Cladomorphinae (MLB = 94, BPP = 0.98), Lanceocercata (MLB = 97, BPP = 0.98), Lonchodinae (BPP = 0.97), Pharnaciini (MLB = 75, BPP = 0.99), Phylliinae (MLB = 100, BPP = 0.99) and Stephanacridini (MLB = 98, BPP = 0.99). Clades less well supported, but also monophyletic in accordance with previous studies [24, 30–32] are Heteropteryginae (BPP = 0.93), Necrosciinae (BPP = 0.94), Pseudophasmatinae (BPP = 0.91) and Diapheromerinae (BPP = 0.63). Some further, previously unrecognized groupings that receive good support comprise a Malagasy lineage *Achrioptera* + Anisacanthidae + Damasippoididae (MLB = 61, BPP = 0.98) and an African clade of paraphyletic Gratidiini (*Gratidia*, *Zehntneria*) + *Phalces* (Bacillinae) (MLB = 100, BPP = 0.98). Bacillidae (*Antongilia*, *Phalces*, *Xylica*) and Pachymorphinae (*Clonaria, Gratidia, Pachymorpha, Sceptrophasma, Spinotectarchus, Zehntneria*) were recovered as polyphyletic.

Within Lanceocercata we corroborated earlier findings that Platycraninae is monophyletic (MLB = 100, BPP = 1) including *Graeffea seychellensis*, but excluding *Apterograeffea*, and a clade comprising all taxa from New Caledonia and New Zealand (MLB = 54, BPP = 0.98) [20, 24, 33]. All individuals sampled from the Mascarene archipelago (9 species, 6 genera) form one strongly supported clade (MLB = 98, BPP = 0.99) among Lanceocercata. Within the Mascarene lineage, all genera are well supported (MLB = 100, BPP = 1) with a clade of *Monandroptera* + *Rhaphiderus* (MLB = 100, BPP = 1) being sister to the remaining Mascarene taxa (MLB = 100, BPP = 1). *Mauritiophasma* and *Epicharmus* are sister taxa (MLB = 71, BPP = 0.99) as well as *Apterograeffea* and *Monoiognosis* (BPP = 0.87).

The Mascarene clade diverged from the remaining Lanceocercata approximately 27.15 mya, between 20.16 and 33.07 mya, and started diversifying around 22.03 mya ago, between 15.99 and 29.39 mya. The overall divergence time estimates indicate a recent diversification of Euphasmatodea beginning ~61.26 mya (51.04–75.43 mya). The split between *Timema* and Euphasmatodea occurred ~103 mya (85.52–122.12 mya).

## Discussion

### Mascarene stick insect phylogeny

Recent studies have revealed that convergently evolved features have often been the basis of classification [24, 34]. Molecular studies provide a breakthrough in the investigation of these radiations since they avoid the use of circularly interpreted characters. Our molecular phylogeny did not support the traditional classification of Mascarene stick insects, which predicted that the respective fauna consists of five unrelated lineages, and, consequently, implied the occurrence of multiple colonisation events leading to the present-day phasmatodean community in the archipelago [22]. Instead we recovered a single origin of all Mascarene stick insects, forming a strongly supported lineage deeply nested within the highly diverse Australasian Lanceocercata (Fig. 2). All Mascarene genera we sampled here (6) and the majority of species (7) are found on Mauritius, which is generally considered to be the oldest island in the archipelago, its volcanic emergence dated 7.8 mya [5]. Three genera, *Apterograeffea*, *Monandroptera*, and *Rhaphiderus*, also have species on Réunion, which is a much younger island dated at 2.1 mya [5]. The age of Rodrigues has been debated with estimates ranging between 11 mya for the old volcanic ridge and 1.5 mya for the recent emergent part [5], some sources give 15 mya as the oldest date of its emergence [7, 8, 14]. The only reported, endemic stick insect from Rodrigues, *Xenomaches incommodus* (Butler, 1876), which is an extinct member of the Platycraninae [35], was not available for our analysis.

The Lanceocercata clade has undergone an impressive evolutionary diversification during the past ~40 million years that led to several adaptive radiations in geographical isolation, e.g., New Caledonia and New Zealand, resulting in several morphologically and behaviourally convergent ecotypes [20, 24, 33]. Therefore, it did not come as a surprise that the discovery of Lanceocercata rendered numerous traditional systematic entities artificial. Lanceocercata comprises species from six subfamilies, Pachymorphinae, Tropidoderinae, Xeroderinae, Phasmatinae, Eurycanthinae, and Platycraninae, none of which is monophyletic. This knowledge is significantly enhanced by our finding of another unexpected adaptive radiation in the Mascarene archipelago, which involves further strikingly convergent ecotypes. For instance, the Mascarene *Apterograeffea* (Fig. 1a), traditionally viewed as a member of the Platycraninae (coconut stick insects and allies), exhibits the same enlarged genae (‘cheeks’) necessary to accommodate the massive mandibular muscles necessary for feeding on hardy palm or screw pine (*Pandanus*) leaves as do the unrelated Australian and Pacific ecotypes, e.g., *Graeffea* and *Megacrania* [22]. On Mauritius, *Epicharmus* (Fig. 1b), traditionally assigned to Xeroderinae, is a stout winged stick insect with lobes on legs and abdomen, which enhance crypsis of this bark-dwelling species, is most reminiscent to the unrelated Australian *Xeroderus* and New Caledonian *Leosthenes*. The female of *Rhaphiderus* (Fig. 1c), hitherto assigned to Tropidoderinae, is a wingless leaf-imitating form very similar to the Australian *Malandania* and *Tropidoderus*. This remarkable diversity in appearance and ecology prompted taxonomists to assume the Mascarene species to be unrelated to each other, especially in regard of the young age of Mauritius, and to incorrectly place them in different subfamilies.

### Origins of the Mascarene fauna

Usually, islands are colonised from the nearest land source, and not surprisingly the Mascarene biota shows close affinities to Madagascar, which is approximately 700 km away. Terrestrial vertebrates like giant tortoises [36], *Phelsuma* day geckos [10] and slit-eared skinks [11] and also recently studied invertebrates such as carnivorous land-snails [12] and the golden orb spider *Nephila* [13] are few examples among many others [7, 8, 14]. We, in contrast, found no close relationships between any Malagasy lineage of stick insects and those of the Mascarene archipelago and, based on a more restricted sampling, already demonstrated the Mascarene Tropidoderinae, *Monandroptera* and *Rhaphiderus*, are deeply nested within the Australasian Lanceocercata [20, 24]. Indo-Pacific ancestors of Mascarene lineages have been revealed before. The most famous example might be the extinct dodo (*Raphus*) on Mauritius and the solitaire (*Pezophaps*) from Rodrigues, which form sister taxa with close relatives in Southeast Asia [15, 16]. *Nactus* geckos and skinks of the genus *Leiolopisma* are further examples showing Australasian origins [19]. The arrival of *Leilopisma* skinks in the Mascarenes requires a spectacular transmarine dispersal between 5600 km from West Australia or more than 7000 km from a more tropical region such as New Guinea. A similar route must have been taken by the ancestral Mascarene stick insect. An east–west directed ocean stream towards Madagascar, the South Equatorial current, which was already existent during the Oligocene and Miocene, is most likely responsible for these kinds of long-distance transoceanic passages [37, 38]. It has recently been demonstrated that the hard-shelled, seed-like eggs of certain phasmatodeans can survive a significant amount of time (several months) under marine conditions, e.g., floating on seawater [39]. Eggs of Mascarene stick insects are often glued to twigs and other plant substrate [23], which would further facilitate above-water dispersal. It is noteworthy that the remarkable transoceanic journey via east–west dispersal across the Indian Ocean occurred twice within Lanceocercata and is not restricted to the Mascarene clade alone. *Graeffea seychellensis*, a member of the true coconut stick insects (Platycraninae) (cf. Fig. 2) that are distributed in Australia and throughout the Southwest Pacific, reached and colonised the Granitic Seychelles. Ocean currents are also likely responsible for repeated dispersals from Mauritius towards Réunion. Both islands have never been connected by land and are separated by 164 km from each other [8], nevertheless members of three Mascarene genera, *Apterograeffea*, *Monandroptera*, and *Rhaphiderus*, colonised Réunion since its emergence. Island colonisations are contingent events. Whereas colonisations of New Caledonia and New Zealand led to flourishing stick insect radiations [20, 24], the Pacific Galápagos Islands and the Hawaiian archipelago are devoid of any phasmatodeans. Peculiarly, Madagascar was never successfully colonised by Lanceocercata [20, 40] either from Australia or the Mascarenes, despite being separated from the latter, viz. Réunion, by only 665 km of ocean [8], far less than the 5600–7000 km that separate Australia from the Mascarenes, and it forms a significantly larger target area than the Mascarene Islands or Seychelles. However, the size and structure of the ancient island community of the Mascarene plateau, which might had much larger landmasses than today, especially during sea-level lowstands [8, 17], remains speculative. We cannot preclude that Lanceocercata might have actually arrived on Madagascar, but was not able to persist and diversify because the Malagasy lineage of stick insects, which colonised the island earlier (between 38 and 51 mya), already underwent a flourishing radiation and largely reduced ecological opportunities for any newcomer.

### Timing the Mascarene colonisation

Most surprisingly, we obtained a time estimate for the origin of the Mascarene clade that significantly exceeds the geological ages of the extant emerged islands. At 22.03 mya (15.99–29.39 mya) the Mascarene phasmid lineage began to radiate long before Mauritius is supposed to have emerged (8–10 mya) [5, 8]. This result is largely contrasted by the recent accumulation of molecular phylogenetic studies favouring recent arrival and diversification of terrestrial colonisers, e.g., 3.5–5.1 mya for *Phelsuma* day geckos [10], ~3 mya for slit-eared skinks [11], 1.9–7.4 mya for hermit spiders and 0.8 mya for *Nephila* spiders [13]. Austin and Arnold [19] recovered a divergence time of 17 mya for Mascarene *Leiolopisma* skinks, however this refers to the stem divergence time separating the Mascarene skinks from their most closely related Australian relatives. This might represent an over-estimate, since the age of the branching between the island lineage and the outside relative (the continental sister group) marks the upper time limit of colonisation, because the closest outside relative might be unsampled or extinct [3]. Consequently, the colonisation event could have occurred significantly later. On the other hand, the age of the most recent common ancestor of a multi-species lineage of island organisms marks the lower limit, because it is possible that the ancestral species colonised the island long before the historically known taxa began to diversify (= crown divergence time) [3]. The crown divergence time of *Leiolopisma* skinks, when speciation on Mauritius began, is dated to 3.4 mya [19], thus being concordant with the island’s geological age. A similar pattern is observed in certain flowering plants (Laurales: Monimiaceae) with a stem divergence time of 31.55 mya between *Monimia* (Réunion) and its sister *Palmeria* (Australia) and a crown divergence time of *Monimia* of 1.34 mya [18]. In contrast, the crown divergence time of the Mascarene palm genus *Hyophorbe* is observed to lie between 12 and 14 mya, albeit with a lower 95 % confidence interval boundary falling within the assumed age of the Mascarene Islands [41], unlike our results.

We used a twofold conservative approach to date the Mascarene phasmid lineage, since our analysis is based on fossil calibrations, and any given fossil indicates the minimum time of the taxon in question, and we refer to the crown divergence time, a potential under-estimate. Taking this into consideration, it is possible that the Mascarene clade (and the whole phasmatodean tree) is even older than suggested by our analysis. We calibrated the split between Phasmatodea and Embioptera at ~122 mya, whereas recent studies based on transcriptomic data inferred much older estimates of ~164 mya [29] and even at ~250 mya [42] and (based on a combination of nuclear and mitochondrial markers) at ~260 mya [43] for the same node. In a previous less conservative approach based on published mitochondrial DNA (mtDNA) rates only, we estimated the stem divergence time of 39.5 mya for the Mascarene members of Tropidoderinae alone [20]. However, molecular clock analyses based on mitochondrial genes can overestimate divergence times [44]. For instance the proposed ancient split (~10 mya) between the two Galápagos iguanas based on mtDNA suggested speciation on now-sunken islands of the archipelago [2], which was recently challenged by a study using protein-coding nuclear genes and arriving at a much younger divergence (~4.5 mya) on currently subaerial islands [45].

Confidence in our time estimates as proposed here gains further support by the dates we obtained for the inter-island splits of the three phasmid genera that have members on both Mauritius and Réunion. The split between the two species of *Apterograeffea* is 3.45 mya (1.41–6.02 mya), and 0.05 mya (0.01–0.11 mya) lie between *Rhaphiderus scabrosus* and *R. spiniger*. The split between the two *Monandroptera acanthomera* specimens, which are considered to represent one single species despite their greater genetic distance, is dated 1.4 mya (0.5–2.51 mya). These dates are consistent with dispersal from Mauritius to Réunion following formation of the latter at least 2.1 mya.

**Fig. 3:**
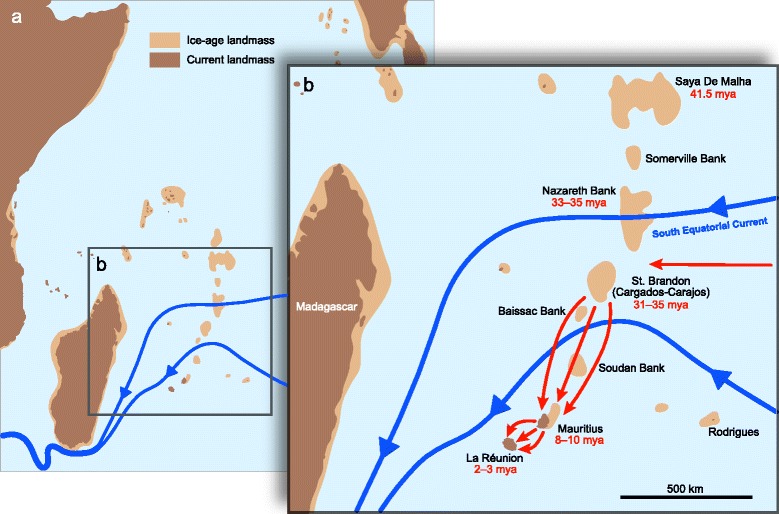
Dispersal scenario of Mascarene stick insects superimposed on a map of the Indian Ocean. **a** Current map of the Indian Ocean. **b** Enlarged view of Mascarene plateau. *Red arrows* indicate postulated colonisation events: The ancestral Mascarene stick insect arrived on currently submerged islands located to the North of Mauritius followed by a radiation and at least three (maximal six) independent dispersals to Mauritius and another three dispersals to Réunion, most likely facilitated by ocean currents (*blue lines*, after [37])

A potential explanation for the discrepancy between the young age of Mauritius and the greater age of the respective lineage of stick insects is provided by historical tectonic and volcanic events in the western Indian Ocean. The Mascarene archipelago is considered to be the product of an age-progressive trend of north-to-south volcanic activity, the Réunion hot spot chain, with a northward increase in age of volcanic activity [5]. It is assumed that many parts of the Réunion hot spot chain were above sea level over the last 65 million years at the time of their formation (Fig. 3) and that these islands would have been temporarily available as stepping-stones for lineages dispersing between India and Madagascar [12, 17]. The next major islands that emerged prior to the rise of Mauritius were Saint Brandon (Cargados-Carajos), 385 km north-east of Mauritius, dated 31 mya, and Nazareth Bank, which is nearly contiguous with St. Brandon bank at its northern margin, dated at 35 mya [8]. Based on our conservative divergence time estimates we reject the possibility of an ancestral Mascarene phasmid arriving in Mauritius and instead propose the scenario of a colonisation of either Nazareth Bank or St. Brandon with subsequent radiation and dispersal towards the much younger Mauritius. Smaller uplifts between St. Brandon and Mauritius, e.g., Baissac Bank and Soudan Bank (Fig. 3), might have further facilitated spread of terrestrial faunal elements. This stepping-stone route was already assumed for the extinct dodo on Mauritius and its sister, the solitaire, on Rodrigues, which exhibit an equally deep divergence time between 23–25 mya [15, 16], and also for two sister taxa of land-snails [12]. With only two taxa involved, the alternative, albeit less parsimonious hypothesis that they diverged on a distant landmass and colonised the islands independently, has to be considered [2]. However, the more diverse radiation of stick insects with a distant Australasian origin makes this scenario very unlikely. It is likewise implausible to assume multiple colonisations from still existent landmasses in the Indian Ocean, such as Madagascar, that requires the assumption that each coloniser’s sister taxon went extinct on this landmass after the colonisation event. Another potential source of colonisation could be Rodrigues whose upper age estimate (15 mya) nearly overlaps with our lower estimate of the Mascarene phasmid clade (16 mya). Given that our estimate, if anything, is an under-estimate, the time-span difference between the origin of the Mascarene clade and the emergence of Rodrigues is probably much larger. Furthermore, past studies have shown that Rodrigues is rather a sink than a source for colonisers with not a single organismic lineage of Rodrigues having colonised any other island in the region [14]. Thus, the possibility of Rodrigues as a source for the Mascarene stick insects appears highly unlikely. A further alternative hypothesis in accordance with our data would be that Mauritius is much older than previously thought. A recent study suggests the presence of a Precambrian microcontinent in the Indian Ocean, now covered by younger magmatic deposits that separated from Madagascar 61–83.5 mya and fragmented into a ribbon-like configuration that also comprises Mauritius [6]. However, this conclusion does not necessarily mean that all of these continental fragments, including Mauritius, were above sea level before the more recent volcanic uplift. Nevertheless, exposed lavas in the Mascarenes are often the result of recent volcanic reactivation and might impede the detection of the true age of the underlying geology [7].

Consequently, we favour the assumption that submerged islands such as St. Brandon have existed continuously above sea level since its formation until the emergence of Mauritius, viz. over a period of more than 20 million years.

### Speciation in the Mascarene archipelago

We found limited evidence for speciation of stick insects on Mauritius. The dates for the inter-generic splits of Mascarene phasmatodeans predate the island’s age again. It is generally assumed that a minimum island size exists below which speciation is rather unlikely due to the lack of opportunities for geographical isolation and niche partitioning [46]. Small islands, like those forming the current Mascarene archipelago, lack speciation in some organismic lineages, as recently observed for *Tetragnatha* spiders [47], because these islands do not allow for sufficient geographical isolation (allopatric speciation) and provide fewer ecological opportunities, e.g., less niche space than large islands, that would more readily allow diversification due to ecological specialisation (sympatric speciation). Yet, the weevil genus *Cratopus* underwent a quite impressive radiation in the archipelago leading to more than 80 species [7]. Before severe habitat destruction since the 17^th^ century, Mauritius was homogenously covered by semi-dry palm forest and moist tropical rain forest [8], but the much younger Mauritius might have been ecologically more diverse. The current landscape of distantly spaced small islands in the Mascarene archipelago was probably preceded by a period of larger islands that more readily allowed for speciation. Therefore, it appears plausible that at least some speciation took place on (intra-island) or between (inter-island) these former, now submerged northern landmasses, which apparently were much larger than Mauritius, e.g., St. Brandon, Nazareth Bank (Fig. 3), followed by repeated colonisations, 3–6 times, of Mauritius. In fact, multiple dispersals towards Mauritius are not unlikely since stick insects already colonised Réunion at least three times during the past 2 million years alone. Allopatry might have played an important role in regard of the deeper nodes of the Mascarene stick insects and was most likely crucial for more recent speciation events. With the exception of *Monoiognosis*, which has only very recently diverged into two species on Mauritius, further intra-generic splits occurred only in geographical isolation after colonisation of Réunion. Similar patterns are observed in the Mascarene radiations of giant tortoises [36] and geckos [10], in which allopatric inter-island speciation as well as sympatric intra-island speciation events are reported.

## Conclusion

In summary, the results of our phylogenetic analysis and divergence time estimates strongly suggest that the Mascarene stick insects are older than the landmass they currently inhabit. We conclude that the ancestral Mascarene stick insect colonised former, now sunken islands, most probably located to the north of Mauritius. It has been suggested before that subsided seamounts throughout the Indian Ocean, when fully above water, could have served as stepping-stones between India and the Mascarenes and would have reduced the distance for oceanic dispersal [12, 14–18, 41]. However, fossil evidence for a subaerial past has so far only been provided for the Paleocene islands in the Ninetyeast Ridge [48]. Concordant with previous conclusions [8, 15, 41], our results imply that ancient islands must have existed permanently above sea level throughout the Oligocene and Miocene. We expect future phylogeographic investigations in the Mascarene archipelago, which still remains a comparatively uncharted study system, to reveal further deep divergence times in support of our observations and explanations.

## Methods

### DNA extraction and phylogenetic analysis

We extracted and sequenced DNA from 38 stick insect specimens and combined these data with 83 previously sequenced taxa, which represent all major lineages currently recognised in Phasmatodea (see Additional file 1: Table S1 for detailed information) [24, 31]. We focussed on a particularly dense sampling of stick insects from the landmasses of the Indian Ocean. We included nearly all known genera and species from Mauritius and Réunion, which represent members of different taxonomic units, e.g., *Apterograeffea* (Platycraninae), *Epicharmus* (Xeroderinae), *Mauritiophasma* (Phasmatinae), *Monandroptera* and *Rhaphiderus* (Tropidoderinae), and *Monoiognosis* (incertae sedis). We were unable to obtain a sample of *Heterophasma multispinosum* Cliquennois and Brock, 2004 from Réunion, another genus currently placed in Tropidoderinae, and of the extinct *Xenomaches incommodus* from Rodrigues, currently assigned to Platycraninae.

We amplified regions of the mitochondrial cytochrome c oxidase subunit I (COI) and II (COII) genes and the nuclear Histone subunit 3 (H3) and ribosomal large subunit RNA gene (28S) using methods described previously [24]. Chromatograms were edited in Geneious 7.1.5 (http://www.geneious.com) [49]. DNA sequences were aligned using Muscle [50] as implemented in Geneious v7.1.5 (http://www.geneious.com [49]). We used GBLOCKS [51] to identify regions in the alignment with a large number of contiguous conserved positions of a minimum length. We used the GBLOCKS server and implemented the “options for a less stringent selection”. The data were partitioned into four character sets; mitochondrial 1^st^ and 2^nd^ codon positions, mitochondrial 3^rd^ codon positions, H3 gene and 28S gene. Model selection was performed separately on each partition using JModelTest v.2.1.3 [52]. We used the AIC to select from a set of 88 models with base frequencies estimated. Likelihoods were calculated on a BioNJ tree that was fixed across models. Phylogenetic analyses were performed in maximum likelihood and Bayesian frameworks using Garli v2.0 [53] and BEAST 1.7.4 [54] respectively. For the Garli analyses we performed 100 bootstrap replicates with 20 random addition replicates per bootstrap. A partitioned model was employed with parameter estimates from JmodelTest. For the Bayesian analyses we used a log Normal distributed relaxed clock model [55] under a Yule speciation prior (10) with the following exponential priors for the nucleotide substitution model parameters; relative substitution rates = 100, relative rate matrix parameters = 1.0, alpha shape parameter = 0.5, uncorrelated log Normal mean = 0.5, uncorrelated log Normal standard deviation = 0.5. For the proportion of invariable sites a uniform distribution bounded by 0 and 1.0 was used. The analyses were run for 60 million generations, sampling every 1000 generations.

### Fossil calibrations

To calibrate the phylogeny we used four fossils that we were able to assign to specific nodes on the tree. The first calibration was obtained from the Yixian Formation in Liaoning, China based on an adult male specimen, *Renphasma sinica* [56]. This is the oldest fossil that can be clearly identified as a true phasmatodean [57]. Males of Phasmatodea possess a sclerotised hook on the venter of abdominal segment 10, the vomer, between a pair of unsegmented cerci, which are interpreted as unambiguous apomorphic characters for Phasmatodea [58], both exhibited by *Renphasma* [56]. The Yixian Formation dates from at least 122.1 mya [59]. We calibrated the root of the tree that separates the Embioptera from the Phasmatodea with an exponential prior with mean of 1.0 and offset at 122 mya. This represents a conservative approach to dating because we have calibrated the deeper Embioptera/Phasmatodea split, rather than shallower crown group radiations. The effect of this conservative approach is to yield more recent divergence times than applying crown-group calibrations. Further calibrations were provided based on fossil eggs. Eggs of stick insects are hard-shelled and richly sculptured with species-specific traits that are considered to be of major taxonomic importance for the order [32, 60]. The second calibration was from Clark Sellick [61], who described fossil eggs from the Eocene Clarno Formation Nut Beds of Oregon. These species can confidently be assigned to the Pseudophasmatinae subgroup Anisomorphini because their eggs bear a conspicuous negative opercular angle [61], which represents a derived state among phasmatodean eggs. The Clarno Formation has been dated to 44 mya [62]. We calibrated the basal node of the Pseudophasmatinae with an exponential prior with mean of 1.0 and offset at 44 mya. The third and fourth calibrations were taken from Poinar [63] who described two fossil eggs from *Clonistria* and *Malacomorpha* from Dominican amber on Hispaniola. We used the *Malacomorpha* fossil to date the divergence of this genus from its sister taxon *Anisomorpha*. Eggs of some *Malacomorpha* species, e.g., *M. cyllarus* (Westwood, 1859), have a very specific rugose capsule with raised net-like protuberances [64]. We used the *Clonistria* fossil to date the basal divergence of the Diapheromerinae. *Clonistria* eggs can be determined by their vesicular, open capitulum broadly covering the operculum, which is not present in the ground pattern of Diapheromerinae (absent in *Ocnophiloidea* and *Lobolibethra*) [60, 63]. Both genera, *Clonistria* and *Malacomorpha* have numerous extant members on Hispaniola [63, 64]. Dating of Dominican amber is controversial, with the youngest proposed age of 15–20 mya based on foraminifera and the oldest as 30–45 mya based on coccoliths [65, 66], with the specific amber containing the fossil phasmatodeans from La Buscara mine being dated between 20 and 40 million years [63]. In order to be conservative we used an exponential prior with mean of 1.0 and offset at 20 and 40 mya respectively.

## Additional file

Additional file 1: Table S1. List of species included in this study with Genbank accession numbers for each gene. Cells with a dash indicate missing data. Taxa in alphabetical order with subfamily assignment following [24] with amendments from [22, 23, 31]. Mascarene taxa in bold. (PDF 167 kb)
