# Supplementary material for: Single origin of the Mascarene stick insects: ancient radiation on sunken islands?
Source: BMC Evol Biol. 2015 Sep 16;15:196. doi: 10.1186/s12862-015-0478-y (PMC4573937; doi:10.1186/s12862-015-0478-y)
Supplement: Additional file 1: Table S1. — List of species included in this study with Genbank accession numbers for each gene. Cells with a dash indicate missing data. Taxa in alphabetical order with subfamily assignment following [24] with amendments from [22, 23, 31]. Mascarene taxa in bold. (PDF 167 kb) [file 12862_2015_478_MOESM1_ESM.pdf]

Table 1. List of species included in this study with Genbank accession numbers for each gene. Cells with a dash indicate missing data. Taxa in alphabetical order with subfamily assignment following (24) with amendments from (22,23,31). Mascarene taxa in bold.

Taxon	Subfamily	Locality	COI	COII	H3	28S
Abrosoma festinatum Brock & Seow-Choen, 1995	Aschiphasmatinae	Malaysia	FJ474257.1	FJ474334.1	FJ474178.1	FJ474100.1
Acanthoclonia sp.	Pseudophasmatinae	Rio Topo, Ecuador	KT426622	KT426593	-	KT426653
Acanthoxyla geisovii (Kaup, 1866)	Phasmatinae	Dunedin, New Zealand	EU492962.1	EU492989.1	FJ474187.1	EU492933.1
Achrioptera punctipes (Audinet-Serville, 1838)	Phasmatinae	Madagascar	FJ474262.1	FJ474338.1	FJ474183.1	FJ474105.1
Acrophylla titan (Macleay, 1826)	Phasmatinae	NSW, Australia	FJ474258.1	FJ474335.1	FJ474179.1	FJ474101.1
Acrophylla wuelfingi (Redtenbacher, 1908)	Phasmatinae	Australia	GQ927385.1	GQ927421.1	GQ927458.1	GQ927347.1
Agathemera crassa (Blanchard, 1851)	Agathemerinae	Chile	FJ474260.1	FJ474337.1	FJ474181.1	FJ474103.1
Agamemnon cornutus (Burmeister, 1838)	Cladomorphinae	West Indies	FJ474261.1	KT426594	FJ474182.1	FJ474104.1
Anchiale briareus (Gray, 1834)	Phasmatinae	Queensland, Australia	FJ474295.1	FJ474369.1	FJ474217.1	FJ474139.1
Anchiale sp.	Phasmatinae	Australia	FJ474270.1	FJ474346.1	FJ474193.1	FJ474113.1
Anisomorpha buprestoides (Stoll, 1813)	Pseudophasmatinae	Florida, USA	FJ474263.1	FJ474339.1	FJ474184.1	FJ474106.1
Antongilia muricata (Redtenbacher, 1906)	Antongiliinae	Périnet, Madagascar	KT426623	KT426595	KT426565	-
***Apterograeffea marshallae* Cliquennois & Brock, 2002**	**Platycraninae**	**Mauritius, île Ronde**	**KT426624**	**KT426596**	**KT426566**	**KT426654**
***Apterograeffea reunionensis* Cliquennois & Brock, 2002**	**Platycraninae**	**La Réunion**	**KT426625**	**KT426597**	**KT426567**	**KT426655**
Asprenas impennis Carl, 1913	Eurycanthinae	New Caledonia	FJ474264.1	FJ474340.1	FJ474185.1	FJ474107.1
Autolyca sp.	Pseudophasmatinae	Panama	KT426626	KT426598	KT426568	KT426656

Bacteria ferula (Fabricius, 1793)	Diapheromerinae	Guadeloupe	FJ474266	KT426599	FJ474188	KT426657
Bactrododema hecticum (Lichtenstein, 1796)	Palophinae	Riefontein, East Cape Province, South Africa	FJ474327.1	FJ474396.1	FJ474249.1	FJ474171.1
Caledoniaphasma marshallae Zompro, 2001	Xeroderinae	Col d'Amieu, New Caledonia	GQ927397.1	GQ927432.1	GQ927470.1	GQ927359.1
Canachus alligator Redtenbacher, 1908	Eurycanthinae	New Caledonia	FJ474267.1	FJ474343.1	FJ474190.1	FJ474110.1
Canachus sp.	Eurycanthinae	Mt Panie, New Caledonia	FJ474269.1	FJ474345.1	FJ474192.1	FJ474112.1
Carlius fecundus (Carl, 1915)	Eurycanthinae	Mt Kohgis, New Caledonia	FJ474265.1	FJ474341.1	FJ474186.1	FJ474108.1
Carausius morosus (Sinéty, 1901)	Lonchodinae	India	FJ474268.1	FJ474344.1	FJ474191.1	FJ474111.1
Chitoniscus brachysoma (Sharp, 1898)	Phylliinae	Plateau de Dogny, New Caledonia	FJ474271.1	-	FJ474194.1	FJ474114.1
Chitoniscus feejeanus (Westwood, 1864)	Phylliinae	Vanua Levu, Fiji	FJ474272.1	FJ474347.1	FJ474195.1	FJ474115.1
Chondrostethus woodfordi Kirby, 1896	Lonchodinae	Guadalcanal, Solomon Islands	FJ474273.1	FJ474348.1	FJ474196.1	FJ474116.1
Cigarrophasma tessellatum Brock & Hasenpusch, 2001	Phasmatinae	Kuranda, Australia	KT426627	KT426600	-	KT426658
Clitarchus hookeri (White, 1846)	Phasmatinae	Peel Forest, New Zealand	EU492968.1	EU492994.1	FJ474189.1	EU492943.1
Clonaria conformans (Brunner, 1907)	Pachymorphinae	Vietnam	FJ474288.1	FJ474363.1	FJ474210.1	FJ474132.1
Cnipsus rachis (Saussure, 1878)	Xeroderinae	Col d'Amieu, New Caledonia	FJ474274.1	FJ474349.1	FJ474197.1	FJ474117.1
Creoxylus spinosus (Fabricius, 1775)	Pseudophasmatinae	Trinidad	-	KT426601	KT426569	KT426659
Damasippoides erythropterus Redtenbacher, 1906	Damasippoidinae	Vohibola, Madagascar	-	-	KT426570	KT426660

Diapherodes venustula (Serville, 1838)	Cladomorphinae	Cuba	KT426628	KT426602	KT426571	KT426661
Diapheromera femorata (Say, 1824)	Diapheromerinae	Kiowa County, Kansas, USA	FJ474276.1	FJ474351.1	FJ474199.1	FJ474119.1
Dimorphodes mancus Bates, 1865	Xeroderinae	Papua New Guinea	FJ474278.1	FJ474353.1	FJ474201.1	FJ474121.1
Dimorphodes sp.	Xeroderinae	Papua New Guinea	FJ474277.1	FJ474352.1	FJ474200.1	FJ474120.1
Dinophasma saginatum (Redtenbacher, 1906)	Aschiphasmatinae	Sabah, Borneo	FJ474279.1	-	-	FJ474122.1
Dryococelus australis (Montrouzier, 1855)	Eurycanthinae	Balls Pyramid, Lord Howe Island, Australia	FJ474280.1	FJ474354.1	FJ474202.1	FJ474123.1
***Epicharmus marchali* (Serville, 1838)**	**Xeroderinae**	**Mauritius**	**KT426629**	**KT426603**	**KT426572**	**KT426662**
Epidares nolimetangere (Haan, 1842)	Heteropteryginae	Borneo	FJ474281.1	FJ474355.1	FJ474203.1	FJ474124.1
Erinaceophasma vepres (Brunner, 1907)	Lonchodinae	Papua New Guinea	-	FJ474356.1	FJ474204.1	FJ474125.1
Eurycantha calcarata Lucas, 1869	Lonchodinae	Papua New Guinea	FJ474282.1	FJ474357.1	FJ474205.1	FJ474126.1
Eurycnema goliath (Gray, 1834)	Phasmatinae	Australia	FJ474286.1	FJ474361.1	FJ474208.1	FJ474130.1
Eurycnema osiris (Gray, 1834)	Phasmatinae	Australia	FJ474283.1	FJ474358.1	FJ474206.1	FJ474127.1
Extatosoma tiaratum bufonium Westwood, 1874	Tropidoderinae	Australia	FJ474285.1	FJ474360.1	-	FJ474129.1
Extatosoma tiaratum tiaratum (Macleay, 1826)	Tropidoderinae	Australia	FJ474284.1	FJ474359.1	FJ474207.1	FJ474128.1
Graeffea seychellensis Ferriere, 1912	Platycraninae	Seychelles	KT426630	-	KT426573	KT426663
Gratidia sp. STI11	Pachymorphinae	Silvermine Nature Res., W. Cape Province,	KT426631	KT426621	KT426592	KT426686

		South Africa				
Gratidia sp. STI15	Pachymorphinae	Burnt Kraal, East Cape Province, South Africa	FJ474333.1	FJ474403.1	FJ474256.1	FJ474177.1
Haaniella scabra (Redtenbacher, 1906)	Heteropteryginae	Borneo	KT426632	KT426604	KT426574	KT426664
Heteropteryx dilatata (Parkinson, 1798)	Heteropteryginae	Malaysia	FJ474291.1	FJ474366.1	FJ474213.1	FJ474135.1
Hyrtacus sp. STI12	Lonchodinae	Tenant Springs, Australia	FJ474326.1	FJ474395.1	FJ474248.1	FJ474170.1
Isagoras sp.	Pseudophasmatinae	Ecuador	KT426633	-	KT426575	KT426665
Labidiophasma rouxi Carl, 1915	Eurycanthinae	New Caledonia	FJ474297.1	FJ474371.1	FJ474219.1	FJ474141.1
Lamponius sp.	Cladomorphinae	Puerto Rico	KT426634	KT426605		KT426666
Leiophasma sp.	Leiophasmatinae	Madagascar	KT426635	KT426606	KT426576	KT426667
Leosthenes sp.	Xeroderinae	Aoupinie, New Caledonia	FJ474303.1	FJ474377.1	FJ474225.1	FJ474147.1
Lobolibethra mainerii (Giglio-Tos, 1910)	Diapheromerinae	Peru	-	-	-	KT426675
Macrophasma biroi (Redtenbacher, 1908)	Phasmatinae	Papua New Guinea	FJ474289.1	FJ474364.1	FJ474211.1	FJ474133.1
Malacomorpha cyllarus (Westwood, 1859)	Pseudophasmatinae	Jamaica	KT426636	KT426607	KT426577	KT426668
Malandania pulchra Sjöstedt, 1918	Tropidoderinae	Kuranda, Australia	FJ474293.1	FJ474367.1	FJ474215.1	FJ474137.1
***Mauritiophasma motalai* Cliquennois & Brock, 2004**	**Phasmatinae**	**forêt de Brise Fer, District de Plaine Wilhems, Mauritius**	**-**	**KT426608**	**KT426578**	**KT426669**
Medauroidea extradentata (Brunner, 1907)	Clitumninae	Vietnam	KT426637	-	KT426579	KT426670
Megacrania alpheus Kirby, 1896	Platycraninae	Australia	FJ474298.1	FJ474372.1	FJ474220.1	FJ474142.1
Megacrania phelaus (Westwood,	Platycraninae	Malaita,	FJ474299.1	FJ474373.1	FJ474221.1	FJ474143.1

1859)		Solomon Islands				
Metoligotoma sp.	Australembiinae	Australien	EU157065.1	EU157073.1	EU157033.1	EU157054.1
Metriophasma iphicles (Redtenbacher, 1906)	Pseudophasmatinae	Panama	-	KT426609	-	KT426671
Microcanachus matileorum Donskoff, 1988	Eurycanthinae	Mt Panie, New Caledonia	FJ474300.1	FJ474374.1	FJ474222.1	FJ474144.1
***Monandroptera acanthomera* (Burmeister, 1838) 1**	**Tropidoderinae**	**Mauritius**	**FJ474301.1**	**FJ474375.1**	**FJ474223.1**	**FJ474145.1**
***Monandroptera acanthomera* (Burmeister, 1838) 2**	**Tropidoderinae**	**La Réunion**	**FJ474302.1**	**FJ474376.1**	**FJ474224.1**	**FJ474146.1**
***Monoiognosis bipunctata* Cliquennois & Brock, 2004**	***incertae sedis***	**Montagne Cocotte, Mauritius**	**KT426638**	**KT426610**	**KT426580**	**KT426672**
***Monoiognosis spinosa* Cliquennois & Brock, 2004**	***incertae sedis***	**Montagne Cocotte, Mauritius**	**KT426639**	**KT426611**	**KT426581**	**KT426673**
Necrosciinae sp.	Necrosciinae	Queensland, Australia	FJ474304.1	FJ474378.1	FJ474226.1	FJ474148.1
Neophasma subapterum Redtenbacher, 1906	Pseudophasmatinae	Venezuela	KT426640	-	KT426582	KT426674
Neopromachus doreyanus (Bates, 1865)	Lonchodinae	Papua New Guinea	FJ474305.1	FJ474379.1	FJ474227.1	FJ474149.1
Nov. gen. 1, nov. sp. 1	*incertae sedis*	Plateau de Dogny, New Caledonia	GQ927393.1	GQ927429.1	GQ927466.1	GQ927355.1
Ocnophiloidea sp.	Diapheromerinae	Peru	KT426641	KT426612	-	KT426676
Onchestus rentzi Brock & Hasenpusch, 2006	Phasmatinae	Kuranda, Australia	KT426642	KT426613	KT426583	KT426677
Oreophoetes peruana (Saussure, 1868)	Diapheromerinae	Peru	KT426643	KT426614	AY125236.1	-
Orxines xiphias (Westwood, 1859)	Necrosciinae	Philippines	FJ474306.1	-	FJ474228.1	FJ474150.1
Oxyartes lamellatus Kirby, 1904	Necrosciinae	Vietnam	FJ474307.1	FJ474380.1	FJ474229.1	FJ474151.1

Pachymorpha sp.	Pachymorphinae	Queensland, Australia	FJ474308.1	FJ474381.1	FJ474230.1	FJ474152.1
Parectatosoma mocquerysi Finot, 1898	Anisacanthinae	Madagascar	KT426644	KT426615	KT426584	KT426678
Parapodacanthus hasenpuschorum Brock, 2003	Tropidoderinae	Kuranda, Queensland, Australia	KT426645	KT426616	KT426585	KT426679
Paronchestus charon Redtenbacher, 1908	Phasmatinae	Alpha, Queensland, Australia	KT426646	KT426617	KT426586	KT426680
Peruphasma schultei Conle & Hennemann, 2005	Pseudophasmatinae	Peru	FJ474309.1	-	FJ474231.1	FJ474153.1
Phaenopharos khaoyaiensis Zompro, 2000	Necrosciinae	Thailand	FJ474313.1	FJ474383.1	FJ474235.1	FJ474157.1
Phasmotaenia lanyuhensis Huang & Brock, 2001	Phasmatinae	Taiwan	KT426647	KT426618	KT426587	KT426681
Phalces tuberculatus Brock, 2000	Bacillinae	South Africa	KT426648	KT426619	-	KT426682
Pharnacia ponderosa Stål, 1877	Phasmatinae	Philippines	FJ474314.1	-	FJ474236.1	FJ474158.1
Phasma gigas (Linnaeus, 1758)	Phasmatinae	Papua New Guinea	FJ474385.1	FJ474316.1	FJ474238.1	FJ474160.1
Phasmotaenia inermis (Redtenbacher, 1908)	Phasmatinae	Fiji	GQ927383.1	GQ927419.1	GQ927456.1	GQ927345.1
Phasmotaenia spinosa Hennemann & Conle, 2001	Phasmatinae	Malaita, Solomons	FJ474310.1	FJ474382.1	FJ474232.1	FJ474154.1
Phobaeticus kirbyi Brunner, 1907	Phasmatinae	Keningau, Sabah, Borneo	KT426649	-	KT426588	-
Phyllium celebicum Haan, 1842	Phylliinae	Thailand	FJ474311.1	-	FJ474233.1	FJ474155.1
Phyllium giganteum Hausleithner, 1984	Phylliinae	Malaysia	-	-	FJ474234.1	FJ474156.1
Phyllium siccifolium (Linnaeus, 1758)	Phylliinae	Philippines	FJ474315.1	FJ474384.1	FJ474237.1	FJ474159.1
Podacanthus wilkinsoni Macleay,	Tropidoderinae	Australia	FJ474317.1	FJ474386.1	FJ474239.1	FJ474161.1

1882						
Pseudoleosthenes irregularis Redtenbacher, 1906	Damasippoidinae	Vohimana, Madagascar	KT426650	-	KT426589	KT426683
Pseudosermyle phalangiphora (Rehn, 1907)	Diapheromerinae	Belize	FJ474318.1	FJ474387.1	FJ474240.1	FJ474162.1
Pseudophasma velutinum (Redtenbacher, 1906)	Pseudophasmatinae	Peru	FJ474319.1	FJ474388.1	FJ474241.1	FJ474163.1
Pterinoxylus crassus Kirby, 1899	Cladomorphinae	Guadeloupe	FJ474320.1	FJ474389.1	FJ474242.1	FJ474164.1
Pterobrimus depressus Redtenbacher, 1906	Heteropteryginae	Viti Levu, Fiji	FJ474287.1	FJ474362.1	FJ474209.1	FJ474131.1
Ramulus thaii Hausleithner, 1985	Clitumninae	Thailand	FJ474322.1	FJ474391.1	FJ474244.1	FJ474166.1
***Rhaphiderus scabrosus* (Percheron, 1829-1838)**	**Tropidoderinae**	**Mauritius**	**FJ474321.1**	**FJ474390.1**	**FJ474243.1**	**FJ474165.1**
***Rhaphiderus spiniger* (Lucas, 1862)**	**Tropidoderinae**	**La Réunion**	**KT426651**	**KT426620**	**KT426590**	**KT426684**
Rhynchacris ornata Redtenbacher, 1908	Cladomorphinae	Costa Rica	FJ474292.1	-	FJ474214.1	FJ474136.1
Sceptrophasma hispidulum (Wood-Mason, 1873)	Pachymorphinae	Andaman Islands	FJ474323.1	FJ474392.1	FJ474245.1	FJ474167.1
Sipyloidea sipylus (Westwood, 1859)	Necrosciinae	Singapore	FJ474324.1	FJ474393.1	FJ474246.1	FJ474168.1
Spathomorpha adefa Cliquennois, 2005	Phasmatidae *incertae sedis* or Antongiliinae[32]	Périnet, Madagascar	KT426652	-	KT426591	KT426685
Spinohirasea bengalensis (Brunner, 1907)	Necrosciinae	Vietnam	FJ474325.1	FJ474394.1	FJ474247.1	FJ474169.1
Spinotectarchus acornutus (Hutton, 1899)	Pachymorphinae	Okura, Auckland	GQ927403.1	GQ927438.1	GQ927476.1	GQ927365.1
Spinonemia chilensis (Westwood, 1859)	Heteronemiinae	Vicente Perez Rosales, Peulla, Chile	FJ474290.1	FJ474365.1	FJ474212.1	FJ474134.1
Thaumatobactron guentheri Hennemann & Conle, 1997	Lonchodinae	Papua New Guinea	-	FJ474397.1	FJ474250.1	FJ474172.1

Timema knulli Strohecker, 1951	Timematinae	California, USA	AY311411.1	DQ241799.1	AY125246.1	AY125302.1
Trachyaretaon carmelae Lit & Eusebio, 2005	Heteropteryginae	Philippines	FJ474329.1	FJ474399.1	FJ474252.1	FJ474174.1
Trapezaspis sp.	Eurycanthinae	New Caledonia	FJ474328.1	FJ474398.1	FJ474251.1	FJ474173.1
Tropidoderus childrenii (Gray, 1833)	Tropidoderinae	Queensland, Australia	FJ474296.1	FJ474370.1	FJ474218.1	FJ474140.1
Vetilia thoon Stål, 1877	Phasmatinae	Queensland, Australia	FJ474294.1	FJ474368.1	FJ474216.1	FJ474138.1
Xeroderus sp.	Xeroderinae	Kuranda, Queensland, Australia	FJ474330.1	FJ474400.1	FJ474253.1	FJ474175.1
Xylica oedematosa Karsch, 1898	Bacillinae	Tanzania	FJ474331.1	FJ474401.1	FJ474254.1	-
Zehntneria mystica Brunner, 1907	Pachymorphinae	South Africa	FJ474332.1	FJ474402.1	FJ474255.1	FJ474176.1
